# Supplementary material for: Integrated Biological Control of the Sugar Beet Weevil Asproparthenis punctiventris with the Fungus Metarhizium brunneum: New Application Approaches
Source: Pathogens. 2023 Jan 6;12(1):99. doi: 10.3390/pathogens12010099 (PMC9863636; doi:10.3390/pathogens12010099)
Supplement: Supplementary file 1 [file pathogens-12-00099-s001.zip › pathogens-2079772-supplementary.pdf]

Supplementary Materials

Table S1. Specification of the 13 Emigration fields in Lower Austria.

Location	Coordinates °N	Coordinates °E	Field size
1	48.53085	16.25701	2.8 ha
2	48.42645	15.94265	8.7 ha
3	48.37665	15.97945	4.8 ha
4	48.61820	16.07016	11.5 ha
5	48.45516	16.02725	2.9 ha
6	48.50182	16.44697	3.1 ha²
7	48.67081	15.90948	2.2 ha
8	48.46363	15.92849	5.1 ha
9	48.45774	15.95023	14.3 ha
10	48.41962	15.98718	5.2 ha
11	48.54291	15.929738	7.6 ha
12	48.53868	15.931278	3.5 ha
13	48.53787	15.933020	5.0 ha

Table S2. Allele sizes for *Metarhizium* genotypes isolated from trial sites.

MLG	Ma 2049	Ma 2054	Ma 2063	Ma 2287	Ma 327	Ma 195	Species
1	121	x	146	286	196	102	*M. lepidiotae*
2	129	218	136	303	208	148	*M. robertsii*
3	129	218	136	304	206	111	*M. robertsii*
4	131	232	136	303	208	106	*M. robertsii*
5	129	218	136	303	208	143	*M. robertsii*
6	129	239	141	298	211	120	*M. brunneum*
7	129	239	141	298	211	124	*M. brunneum*
8	133	234	156	302	213	111	*M. brunneum*
9	129	218	136	304	208	106	*M. robertsii*
10	129	220	136	304	206	111	*M. robertsii*
11	129	218	136	303	208	156	*M. robertsii*
12	129	218	136	303	208	145	*M. robertsii*
13	129	218	136	303	208	117	*M. robertsii*
Ma 43	129	239	141	300	211	120	*M. brunneum*

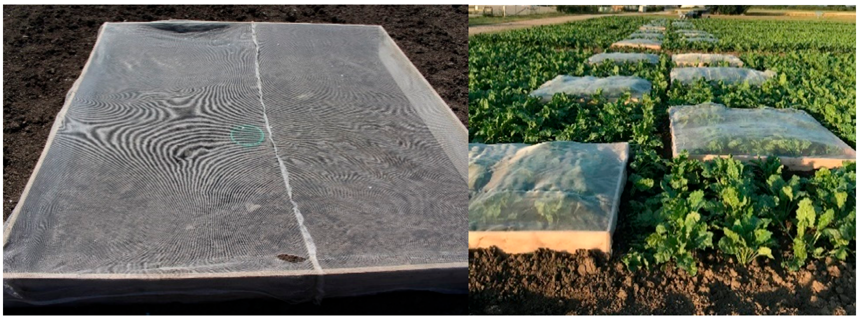

Figure S1. Cages to prevent weevils from leaving the treated plots.

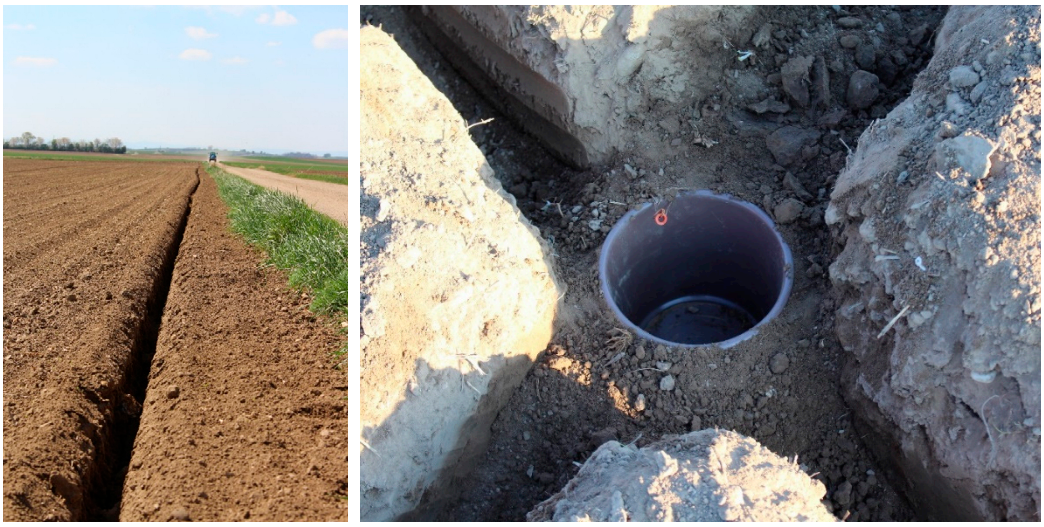

(**A**) (**B**)

Figure S2. Trap ditch (A) and pheromone trap buried in the soil (B) to catch sugar beet weevils.
